# The Power Threat Meaning Framework: a qualitative study of depression in adolescents and young adults

**DOI:** 10.3389/fpsyt.2024.1393066

**Published:** 2024-05-01

**Authors:** Erik Ekbäck, Lina Rådmark, Jenny Molin, Maria Strömbäck, Nick Midgley, Eva Henje

**Affiliations:** ^1^ Department of Clinical Science, Umeå University, Umeå, Sweden; ^2^ Department of Nursing, Umeå University, Umeå, Sweden; ^3^ Department of Community Medicine and Rehabilitation, Umeå University, Umeå, Sweden; ^4^ Department of Clinical, Educational and Health Psychology, University College London, London, United Kingdom

**Keywords:** depression, adolescents, young adults, qualitative research, framework analysis

## Abstract

**Introduction:**

Depression constitutes one of our largest global health concerns and current treatment strategies lack convincing evidence of effectiveness in youth. We suggest that this is partly due to inherent limitations of the present diagnostic paradigm that may group fundamentally different conditions together without sufficient consideration of etiology, developmental aspects, or context. Alternatives that complement the diagnostic system are available yet understudied. The Power Threat and Meaning Framework (PTMF) is one option, developed for explanatory and practical purposes. While based on scientific evidence, empirical research on the framework itself is still lacking. This qualitative study was performed to explore the experiences of adolescents and young adults with depression from the perspective of the PTMF.

**Methods:**

We conducted semi-structured interviews with 11 Swedish individuals aged 15– 22 years, mainly female, currently enrolled in a clinical trial for major depressive disorder. Interviews were transcribed verbatim and analyzed with framework analysis informed by the PTMF.

**Results:**

A complex multitude of adversities preceding the onset of depression was described, with a rich variety of effects, interpretations, and reactions. In total, 17 themes were identified in the four dimensions of the PTMF, highlighting the explanatory power of the framework in this context. Not all participants were able to formulate coherent narratives.

**Discussion:**

The PTMF provides a framework for understanding the complexities, common themes, and lived experiences of young individuals with depression. This may be essential for the development of new interventions with increased precision and effectiveness in the young.

## 1 Introduction

Depression has become the focus of a wealth of research in recent years (1). This is appropriate as major depressive disorder as defined by the Diagnostic and Statistical Manual of Mental Disorders, fifth edition (DSM-5) (2) is predicted to soon be the largest individual contributor to the global burden of disease (1, 3). In adolescence the prevalence of depression increases compared to childhood (4, 5), and an early onset predicts a threefold increase in the risk of adult depression (6) as well as a sixfold increase in all-cause mortality (7). Meta-analyses conclude that available interventions for adolescent depression show some promise but lack clear evidence of efficacy (8–13).

The DSM-5 is based on symptom criteria that largely discounts etiology and the subjective understanding of why one is depressed, which likely contributes to its low diagnostic validity for depressive disorders in this age-group (14–17). A shared understanding of the causes and contributing factors is essential for therapeutic alliance, and subjective causal beliefs seem to influence help-seeking (18). An integrated individual understanding also affects compliance with treatment and ability to handle symptoms (19).

Within the body of depression research there are relatively few qualitative studies that elicit the subjective understanding of the condition, and some previous studies have focused on the lived experience of depression among adolescents (20–25). Some investigators have touched upon the topic of potential causes of depression (23, 26–29) and its treatment (23, 30–37). In summary, the etiology is described as multidimensional, often with more than one cause (23, 26–29). Two studies have applied existing frameworks from psychology/social science (27) as well as physical health contexts (28) in the framing of depression. Still, no one has applied a comprehensive theoretical framework to the lived experience of depression in young people that accounts for both psychological, social, and biological factors without narrowly ascribing primacy to any single one of them.

One framework that could be used to do so is the Power Threat Meaning Framework (PTMF) (38). The PTMF is a complement/alternative to diagnosis-based practice that also aims at the identification of patterns in emotional distress, unusual experiences and troubled or troubling behavior. It is both an over-arching structure for identifying such patterns, and a meta-framework within which existing models and bodies of evidence is accommodated. The framework draws on several philosophical principles, theories, stakeholder perspectives, and scientific evidence. For example, it synthesizes the extensive literature pointing to a causal impact of relational and social adversities on human brain development and a range of emotional outcomes. The PTMF argues that distress, although enabled by and mediated by our bodies and biology, has not in any simplistic sense been shown to be caused by them. As experience is produced and perceived in contexts that are not separate from a range of socially constructed power-structures and social interactions, all imbued with personal narratives and meanings, the PTMF suggests that these aspects need to be integrated in our models to increase their explanatory power (38).

The PTMF may be relevant in the context of depression, which is often associated with profound feelings of powerlessness (22, 30). The PTMF postulates that power is a factor that needs to be considered both in turning current epidemiological trajectories around and to increase treatment effectiveness. An augmented threat-reactivity is furthermore characteristic of adolescent depression, with amygdala hyperreactivity to emotional stimuli (39) and an increased allostatic load (40). If the perceived problems, like e.g., power-imbalances in the affected individuals’ immediate and larger relationships and surroundings (23, 28) or subsequently activated/augmented threat reactions (23) are not addressed, it is likely that the affected individual will feel invalidated. If research is found to support the importance of including these factors, that may invite us to rethink the current interventions for depression in the young and inspire new personalized alternatives with better precision and effectiveness.

The aim of this study was to explore the experiences of adolescents and young adults with depression from the perspective of the PTMF.

## 2 Methods

### 2.1 Study context

The study was conducted with individuals who participated in a multi-center randomized controlled trial (RCT) (41) that investigates the effectiveness of interventions for depression at child and adolescent psychiatric outpatient clinics and youth clinics in two county councils in Northern Sweden. The trial has two phases; first, a one-armed clinical pilot (42) examining the experimental intervention called Training for Awareness Resilience and Action (TARA) (43) and second, an RCT in which participants are randomized to TARA or standard treatment, including but not limited to antidepressant medication and/or psychotherapy. The details of the RCT, including, e.g., eligibility criteria, are outlined in the openly available trial-protocol (41) and online pre-registration (clinicaltrials.gov, NCT-registration identifier: NCT04747340).

### 2.2 Participants

All individuals that had been enrolled in the pilot or RCT at the end of October 2022 were asked to participate in interviews and all agreed to do so (N=66). From this group, nine individuals were randomly selected and interviewed. Two additional individuals were interviewed with purposive sampling to fill remaining knowledge-gaps, resulting in a sample of 10 females and one male, median age 19 years (range 15-23, IQR 4). This extended age range is motivated by neurobiological similarities between adolescents and young adults (44, 45) and recent calls for a new integrated youth mental health care service in this transitional age range (46). All participants had a clinical diagnosis of major depressive disorder, and the mean score on Reynolds adolescent depression self-rating scale 2^nd^ edition (47) was 79.64 (SD 13.02). This scale has good validity and reliability in similar clinical samples and in the Swedish language (48). All participants were Swedish citizens, one was born in the U.S.A., and six had one parent born abroad (from England, Ghambia, India, Iran, Thailand, and the U.S.A.). None reported being part of any of the official national minorities in Sweden.

TARA participants were interviewed before TARA as the intervention has components that are related to the content of the interviews, and standard treatment participants were interviewed either before, during or after treatment.

### 2.3 Procedure and data collection

Ethical approval was obtained from the national ethical review board, (Dnr 2020-05734 and 2021-06418-02) and all participants had provided written informed consent at the time of consenting for the clinical trial. Additional parental consent was not recommended as mandatory by the national ethical review board, and it was therefore not collected. Selected participants were contacted over the phone for more detailed information and oral consent for the interviews. Participation was voluntary and could be terminated at any time. Interviews were conducted in 2022-2023.

Three interviews were conducted by EE and two by a psychology student, all in person at the participants choice of location, most commonly in private rooms at the university or healthcare centers. Six interviews were conducted by LR through a secure online video platform. A semi-structured interview guide was used, with open-ended questions like, e.g., “I am interested in knowing more about what you think caused or contributed to your depression, what do you think?”. Follow-up questions informed by the PTMF-dimensions were asked to encourage participants to develop their narratives. For example, “Have you experienced anything that made you very scared or powerless?”, and “How did that affect you?”. More open follow-up questions, like “What other factors may have been important in your case?” were also included.

After six interviews the interview guide was modified to better probe different aspects of perceived causality and more details of treatment(s) received. Interviews lasted for 18-90 min (median 74 min) and the shortest one was ended prematurely by the participant due to an emerging painful reluctancy. All participants received a compensation equivalent to 20 Euro. Interviews were audio-recorded and transcribed verbatim.

### 2.4 Analysis

Data was analyzed using framework analysis (49–52) to elicit the participants’ experiences of depression based on the dimensions of the PTMF. Framework analysis allows researchers to both bring a pre-defined set of issues and be responsive to emerging themes.

Transcripts were read by all five analysts for familiarization, and emergent issues were discussed to get an overall understanding of the data. The text was then divided and condensed into meaning units relevant to the aim of the study, and the meaning units were coded and sorted according to the dimensions of the PTMF (Power, Threat, Meaning, and Threat Response). These steps were performed individually by EE and LR who then compared their work and adjusted as necessary. All analysts then provided input on the sorting and agreed upon the format. The codes in each dimension were then grouped, abstracted, and interpreted into themes. All analysts met regularly in reflective dialogues to discuss interpretations and finally agreed on the structure and content of the results. Analysts were familiar with the PTMF prior to the analysis. The software MAXQDA 22.8.0 (2022, VERBI software GmbH, Berlin Germany) was used, and no AI tools were applied. The Consolidated Criteria for Reporting Qualitative Research (COREQ) (53) guided this report.

## 3 Results

The results are presented as 17 themes, all incorporated into the four dimensions of the PTMF, to describe the participants experiences of their depression. An overview of the results is presented in **Table 1**. An elaboration of the results is presented in text and codes are compared within and across framework dimensions and themes. Quotations are included to illustrate themes and support analytical claims. The quotations have been translated from Swedish to English [translator’s clarifications are bracketed].

**Table 1:** Findings based on the PTMF-dimensions.

Dimensions of the PTMF (and corresponding questions)	Themes
Power (What has happened to you)?	Loss or fear of loss Parental distress and lack of family support Social exclusion and stress Abuse and harassment Invalidated by the health care system
Threat (How did that affect you)?	Getting shocked and confused Getting angry and frustrated Experiencing anxiety and physical symptoms Feeling low and lonely
Meaning (What sense did you make of it)?	Feeling left out Lacking trust in others and in oneself Blaming oneself
Threat response (What did you have to do to survive)?	Keeping things within and avoiding feelings Withdrawing from relationships and coping alone Self-harming and restrictive eating Seeking contact and support Trying to please and adapt

PTMF, Power Threat Meaning Framework.

### 3.1 Power

This dimension of the PTMF corresponds to questions like “What has happened to you?” and “How has power operated in your life?”. Participants described a range of conditions as potentially related to their depression, and our interpretation of the findings in this dimension is elaborated in the five following themes.

#### 3.1.1 Loss or fear of loss

Loss was described as a cause for depressive symptoms, e.g., losing community or friends due to moving. Participants also expressed a loss of safety, routines and contact with close ones, e.g., due to parental divorce or the birth of a sibling. Natural changes to the body due to puberty, and diseases or injuries with sometimes permanent sequelae were said to make it hard to exercise or hang out with friends in ways that participants were used to. Participants further mentioned disappearances and deaths, sometimes violently, of family members, close friends and/or pets, and expressed fear that other loved ones would also die.

*“I had five rough years where I just lost friend after friend, suicide, diseases, and even a heart attack.” (Participant 5)*

#### 3.1.2 Parental distress and lack of family support

Participants described stressful and unsafe home environments with parents who were busy and preoccupied with their own problems and who did not have the time to help them, engage with them, or even listen to them. Some parents were said to not understand participants’ feelings or the severity of their condition, and some had argued that there was no need for the participant to see a psychologist or seek help in other ways.

*“Home was never really a safe place. Mom was super sick, very depressed, drank a lot, and took a lot of pills. And dad was super stressed from just taking care of her.” (Participant 2)*

Some said their parents struggled with addictions, mental disorders, poverty, and physical disorders. Authoritarian parents were also described, with rigid rules that restricted the families’ and participants’ lives. Some parents were said to be angry with them for being inactive/low, and one participant was kicked out of home. Some participants assumed much responsibility at home, e.g., for siblings or even their parents when they could not cope.

#### 3.1.3 Social exclusion and stress

Social exclusion and stress were common topics, and these were often described as persisting common parts of the participants everyday lives. The participants were often bullied in school both by students, teachers, and other school staff. Social media was also described as a stressful platform for social comparison and competition.

*“And when they said all those mean things, I started to realize it myself too. I started to think that I was the problem.” (Participant 7)*

Some said it was difficult to make new friends even if they wanted to, and therefore they hung out with people despite knowing they were being used by them. There were descriptions of being misunderstood and let down, and not having adults in school that could be trusted. Participants who got in conflict with teachers expressed a lack of support from their peers. Some were struggling with dyslexia, and both before and after receiving this diagnosis participants reported unfair treatment. Additionally, a pressure related to the achievement of high grades was said to come from both teachers, parents, and/or themselves. Outside school hours participants described being left behind as family members took part in social activities without them.

*“I had to stay at home and the whole family went away, so I was all alone in the weekends too.” (Participant 9)*

#### 3.1.4 Abuse and harassment

Previous and ongoing domestic violence was described, including parental assaults directed to the participants themselves, other adults, siblings and/or pets. Participants described parents and/or stepparents who were aggressive and unpredictable in subjecting the participants and/or others to physical and verbal violence, silencing, withholding of information, and who made uninvited intrusive contact. Destructive romantic relationships and sexual abuse was also described, with examples of being forced to do things without consent and being physically hindered to scream for help.

*“If I said no, I knew he would get angry with me, because he got angry a lot. Then it was just like if I said no, and he wanted to have sex, then it was like he just pulled my legs anyway as if I had no choice.” (Participant 5)*

Participants also mentioned sexual harassment by strangers on the street, as well as close relationships in which partners and parents had made them feel stupid.

#### 3.1.5 Invalidated by the health care system

Participants described long waiting times in health care, which contributed to feelings of being insignificant and neglected. Participants also expressed that they were not seen, diminished, and even betrayed by professionals, especially in a child- and adolescent psychiatry. Some described that when trying to explain their problems and feelings, professionals did not seem to understand the nature or severity of their condition, and at times they did not even seem interested in their story.

*“Being treated by someone supposed to help you, who doesn’t even know what they’re doing or why they are there, in the end I just sat there in silence.” (Participant 4)*

Participants also expressed experiences of not getting better by previous treatments, including antidepressant medication and psychotherapy, which led to feelings of invalidation and a lack of solutions or hope. Antidepressants were said to be prescribed without a treatment plan or follow-up, and participants described a shortage of information as to e.g., for how long they would continue the medication.

### 3.2 Threat

This dimension of the PTMF corresponds to questions like “How did that affect you?”. Participants described a variety of ways in which they were affected by being subjected to the different forms of power dynamics described in the previous section, and our interpretation of the findings in this dimension is elaborated in the four following themes.

#### 3.2.1 Getting shocked and confused

Participants described being shocked by things that had happened to them, and this was often accompanied by a struggle to understand their situation and their feelings. Unexpected events were said to trigger disbelief and denial. Situations where participants were lacking an understanding of how they got to a particular place or situation were also described, and so was derealization and movie-like experiences. All this was said to raise more questions that mostly ended up leaving participants confused.

*“Then when he passed away it was like a piece of me followed him. A piece of me that I knew, disappeared completely. Then there I was, I felt a bit empty, but then also so unreal.” (Participant 4)*

#### 3.2.2 Getting angry and frustrated

Based on being fed up with how things were, participants expressed that irritation and anger were common in their lives. Participants described quarrels, a lot of fighting and screaming, both with parents and friends. Often all persons involved were said to be irritated, which led to misunderstandings and further problems. Both overt hatred and a quieter disappointment was described.

*“Everyone said I just got more and more angry, and I had to start seeing a therapist because they said I was having problems with aggression, for taking it out on other things I mean.” (Participant 7)*

#### 3.2.3 Experiencing anxiety and physical symptoms

Negative events were said to trigger fears and expectations that more negative events would occur. This led to worry, fear, social anxiety, and panic attacks that caused avoidance of triggering places and situations. A tendency to overthink and over-analyze was also described, and this was said to make it difficult to sleep or get other important things done.

*“I just can’t relax; I must analyze how the person reacted. And I have trouble sleeping from having to go through the entire day before I go to sleep.” (Participant 2)*

Physical symptoms like muscular tension and pain were common, and nausea, palpitations, a heaviness in the chest, shortness of breath, lumps in the throat or sense of being strangulated, hair loss, headaches, and stomachaches were described. Previous experiences were also said to have made participants more alert, sensitive, and easily triggered by things, even small insignificant sounds. One participant got really scared if someone sneezed. Participants reported that they vigilantly observed previous perpetrators, often in their home, for indicators of their current mood. If the person raised their voice, touched them, or even stayed silent it was said to indicate that more negative things were coming.

#### 3.2.4 Feeling low and lonely

As a result of negative operations of power, participants described getting tired and low, with little or no energy to engage in their everyday lives. Things they used to enjoy were said not to feel as enjoyable. Some said it was hard to start new projects when feeling sick, leading to procrastination. Further descriptions included apathy, depression, and a deep sadness. A sense of loneliness was also common and moreover a “stuck-ness” with oneself and an inability to reach out to others.

*“In the beginning I didn’t notice it much, it was more like I just didn’t have any energy to do stuff, it was no longer fun to do things I used to like.” (Participant 8)*

### 3.3 Meaning

This dimension of the PTMF corresponds to questions like “What sense did you make of it?”. Participants described different ways of understanding their situation, and a few participants expressed that they were still struggling to make sense of the meaning of some events. Our interpretation of the findings in this dimension is elaborated in the three following themes.

#### 3.3.1 Feeling left out

Participants sometimes interpreted their situation as if they did not belong anywhere. They described that they had nothing in common with the people around them, and therefore had a sense of not fitting in. By not understanding the social codes or themselves in relation to others, and perceiving themselves as embarrassing and in the way, participants expressed that they lost their groups and sense of community and belonging.

*“I have nothing in common with them, they laugh at things I don’t understand. I mean it is like I’ve been living underneath a stone my entire life.” (Participant 11)*

#### 3.3.2 Lacking trust in others and in oneself

Participants described insecurities, doubt, and mistrust in others and in themselves. They expressed feeling such as no one could help them and therefore found it hard to rely on others. Others were said not to care. This, and the experience of losing others - which was often interpreted to mean that “goodbye means bye forever” - made some participants draw the conclusion that they were meant to be alone with their problems.

*“It becomes a defense mechanism or whatever you call it. I mean the trust you have, in my case to my mom particularly. The thing is now, I can’t trust her the way I did before.” (Participant 1)*

On top of that an insecurity regarding their own capability to handle things was expressed. Based on a sense of being wrong, an inability to understand and improve their situation, and not feeling good enough, some were left with a mistrust in their own feelings and thoughts. One said she could not be herself, and others said it was hard to do anything at times of uncertainty.

#### 3.3.3 Blaming oneself

The participants expressed that they assumed responsibility for many things that had happened to them and/or their friends and family.

*“I felt like a failure, inadequate. When I couldn’t fix my parents relationship, and the divorce, like, how was I gonna handle the rest of my life?” (Participant 8)*

After situations of abuse a sense of self-disgust was described, and participants blamed themselves for letting perpetrators do things with them. This was also true with bullying. Seeing faults in oneself was said to result in a bad conscience and seeing oneself as a burden, and failures were described, like e.g., not being able to prevent the death of others, and not being able to explain their situation in a way that health care could understand. Others reported that they created problems out of nothing, that they just exaggerated the problem and overreacted. One said it was like they had “a ghost in the brain”, and others said that if it wasn’t for them problems wouldn’t have happened.

### 3.4 Threat response

This dimension of the PTMF corresponds to questions like “What did you have to do to survive?”. Participants reflected on a variety of coping strategies that they used to handle their situation, and our interpretation is elaborated in the five following themes.

#### 3.4.1 Keeping things within and avoiding feelings

Participants described that they often handled their situation by not talking about problems, sometimes to spare their friends and family from trouble. It was also said that feelings were kept inside, often in attempts to be carefree and “happy-go-lucky “– which sometimes annoyed people around them. Some said they avoided thinking about the situation altogether, pushing things away or denying them. One said she told her parents the truth all at once and that this was not common for her.

*“When I got to see a child psychiatrist, that’s when my mom really got to understand that I actually had been feeling really bad. I had not let her know about that until then.” (Participant 5)*

#### 3.4.2 Withdrawing from relationships and coping alone

Participants described isolating themselves, as sometimes it was said to be easier to be alone. Refusing to go to school, sometimes for several years, was said to negatively affect their grades. A fear of leaving home was expressed, as home was sometimes the only place where they could feel safe and in control. For some, the isolation was said to be an escape, as getting away was sometimes the only possible thing to do. Some said they hung out with others only on the internet as a way of hiding and protecting themselves.

*“I stopped having contact with my friends in school. I didn’t want to talk to them, I was so ashamed, so I have stopped talking to them completely.” (Participant 6)*

Participants also described things they did on their own to try to accept or improve their situation. Some reflected and made plans, others expressed a need to withdraw to spend time with their thoughts and feelings here and now. Some said they used music, movies, or physical exercise to relax and avoid rumination.

*“Getting back from the walk, I have had the time to think and can handle my emotions better, like is this really my fight? No, then I don’t have to get as offended when they are the ones with problems. I can put it to the side a little.” (Participant 1)*

#### 3.4.3 Self-harming and restrictive eating

Participants expressed an urge to harm themselves in different ways, often as other strategies or means of expression were lacking. Knives, blunt objects, and tools were used to injure oneself. Some smoked large amounts of cigarettes and used alcohol and other drugs for this purpose. Participants also described being preoccupied with food and their weight, sometimes restricting their eating, and sometimes also binging as vengeance to parents who had said they ate too much.

*“That [restricted eating] is something I can control, I guess the other things become less in your face when there is at least something you can control.” (Participant 10)*

#### 3.4.4 Seeking contact and support

Help seeking was described by some participants, including seeing psychologists to discuss the situation and find support. Some also described close relationships as important, and family members, teachers and friends who were described as invaluable support. To actively seek out and protect such supportive individuals was a strategy for some. Romantic relationships were sometimes also described as helpful. While some enjoyed opportunities to share their story with other young people in similar situations, others expressed a value in being surrounded by animals.

*“To hear someone else [here at the youth clinic] explain that it is not all that strange that I think along these lines, it makes things easier somehow.” (Participant 3)*

#### 3.4.5 Trying to please and adapt

Participants described that they avoided conflicts in every possible way and did their best to understand others’ feelings, thoughts and needs in order to please them. Some said they tried to compensate for perceived shortcomings by making others happy and satisfied, no matter the cost, and friends were sometimes bought with money or favors. Additionally, participants said they tried to be normal and inconspicuous to fit in.

*“Something that I have had to learn is to analyze everything that happens around me. To always sense what someone needs or if something is wrong, maybe even before they say they need something.” (Participant 10)*

## 4 Discussion

This study explored the experiences of adolescents and young adults with depression from the perspective of the PTMF (38). While the PTMF is based on research, few studies have investigated it empirically in a clinical context. This is perhaps because it has a high level of complexity, with implications that challenge us to rethink key aspects of current psychiatric care and treatment.

In the present study it was feasible to apply the PTMF in the collection and analysis of data, and the dimension of “power” was clearly identified in the interviews. Data contained readily appearing and clearly traumatizing experiences that have all been previously implicated in depression in young people (54–58). We replicate previous findings of interpersonal problems (23, 26, 27, 29) pressure (23, 29), and loss (28) as central to adolescent depression, their common power-related denominator has however previously been largely unrecognized.

The second dimension of “threat” originally refers to what core human needs are threatened by the described negative operations of power. In our sample participants did not spontaneously identify core human needs as threatened in their responses to questions on how they were affected. Themes in this dimension reflect more autonomic stress and threat reactions. This may be due to the questions which were openly formulated. Participants were also relatively young for an analysis of their situation on this level, and potentially the current depression limited their metacognitive capacity. However, in the dimension of “meaning”, the need to belong was prominent, indicating that the ability to identify needs was present. Perhaps some experiences do cut across framework dimensions and therefore fit in more than one. It was expected that the framework would have areas of better and worse fit, as this has been described in previous framework analyses (49, 52).

Subsequent “threat responses” or coping strategies varied in quality and some participants described self-perpetuating negative spirals, e.g., the threat reaction of isolation led to poor school performance, which in turn led to new spirals of school stress and fear of losing other things. In the analysis it was sometimes challenging to see where one chain of events ended and a new one started, and this is acknowledged by the original authors (38). Also, some participants appeared to suffer from dissociation, and some were unable to form a coherent narrative which complicated the matter. To analyze meaning, form a coherent narrative, and regulate emotions are processes that require a coordination of several high-level processes (59), and in young people with depression the fronto-limbic maturation process is delayed in comparison to healthy individuals (60). This, and the inclusions of participants that had not achieved full integration of previous traumatic experiences, can potentially explain the described difficulties. Furthermore, only negative interpretations/meanings were voiced, which may reflect the participants depressed state. The same may be true for negative views on previous therapists and interventions.

Importantly, we identified different meanings ascribed to apparently similar situations and diagnostic labels, indicating the value of personalized approaches to understand, describe, validate, and treat depression successfully. We foresee that contextual, diagnostic, and biomedical approaches to understand, describe and manage depression will complement each other and lead to improved outcomes over time.

Even within the current diagnostic paradigm, trauma informed approaches to depression treatment may be motivated in young people. In this context the PTMF may be helpful in eliciting adverse experiences that were previously obscured. As there is a growing body of evidence that suggests that difficult lives explain depression better than broken brains (61, 62), treatment approaches that acknowledge this can potentially be more effective than interventions that primarily deal with downstream symptoms. One implication of our findings on the relevance of negative operations of power in the context of depression is that intersectional analyses of these mechanisms may be useful, both to enable individual empowerment, restored self-confidence and better life navigation skills as part of depression treatment. Policy reforms aimed to prevent depression in young people may also benefit from such analyses.

Limitations: The interviews might be considered as brief and few when trying to understand the complex phenomena behind depression. We determined the information density to be high, and the material was rich enough for analysis. Only one participant was male however, which may affect transferability. By bringing the PTFM both to the interview design and data analysis, there an almost inevitable risk of “finding” what was simply assumed a priori. To minimize this risk, we carefully searched for meaning units, codes and themes that did not fit within the PTMF, without any such findings. Also, methodological and epistemological triangulation was used by performing framework analysis (51). This allowed us to draw upon existing knowledge and yet not be limited by it, as both realist and constructionist epistemologies were applied. To our knowledge this is the first time that this approach is applied within clinical psychiatry.

Strengths include that that the study gives voice to young people with depression, a group with limited abilities and opportunities to express themselves and be fully heard. The study constitutes a real-world application of the PTMF in a diverse ethnical sample and may contribute to a paradigm shift in the way we conceptualize and address mental disorders at large.

### 4.1 Conclusion

In the last decades there has hardly been any pragmatic or clinically useful alternative available to DSM diagnosis-based practice, and the biomedical and pharmaceutical approach that follows from it may obscure individual needs and lead to missed opportunities for interventions to meet those needs. The PTMF provides an alternative understanding of depression in young people. It is internally coherent and compatible both with the present qualitative data and with previous studies and models of youth depression. As the negative operation of various forms of power appears to be related to the onset of depression in young people, we echo the United Nations special rapporteur: “*Mental health policies should begin to address power imbalances rather than chemical imbalances*” (63). This approach may improve our understanding of depression and inspire the development of new interventions with increased precision and effectiveness.

## Author’s note

EE is a male resident in family medicine and doctoral student in child and adolescent psychiatry. LR is a female pharmacist with a PhD in clinical neuroscience. JM is a female mental health nurse and associate professor in nursing. MS is a female specialist physiotherapist and associate professor in physiotherapy. NM is a male child psychotherapist and a professor of psychological therapies with children and young people. EH is a female specialist and professor in Child and adolescent psychiatry, she developed the TARA intervention. None of the authors had any therapeutic or other relationship to any of the participants and participants were not given any specific information about the researchers. All authors had experience of qualitative research in this area.

## Data availability statement

The raw data supporting the conclusions of this article will be made available by the authors, without undue reservation.

## Ethics statement

The study was approved by the Swedish national ethical review board, (Dnr 2020-05734 and 2021-06418-02). The study was conducted in accordance with the local legislation and institutional requirements. All participants provided written informed consent. Additional parental consent for participants below 18 years of age was not recommended as mandatory by the national ethical review board.

## Author contributions

EE: Conceptualization, Data curation, Formal analysis, Funding acquisition, Investigation, Methodology, Writing – original draft, Writing – review & editing. LR: Writing – original draft, Writing – review & editing, Conceptualization, Data curation, Formal analysis, Investigation, Methodology. JM: Writing – review & editing, Conceptualization, Formal analysis, Investigation, Methodology. MS: Writing – review & editing, Formal analysis. NM: Methodology, Writing – review & editing. EH: Conceptualization, Formal analysis, Funding acquisition, Investigation, Methodology, Writing – review & editing.
